# Ionic Liquid-Modified Gold Nanoparticle-Based Colorimetric Sensor for Perchlorate Detection *via* Anion−π Interaction

**DOI:** 10.1021/acsomega.2c02078

**Published:** 2022-08-03

**Authors:** Büşra Keskin, Ayşem Üzer, Reşat Apak

**Affiliations:** † Institute of Graduate Studies, Istanbul University-Cerrahpaşa, Avcilar, 34320 Istanbul, Turkey; ‡ Department of Chemistry, Faculty of Engineering, Istanbul University-Cerrahpaşa, Avcilar, 34320 Istanbul, Turkey; § Turkish Academy of Sciences (TUBA), Bayraktar Neighborhood, Vedat Dalokay Street No: 112, Çankaya, 06690 Ankara, Turkey

## Abstract

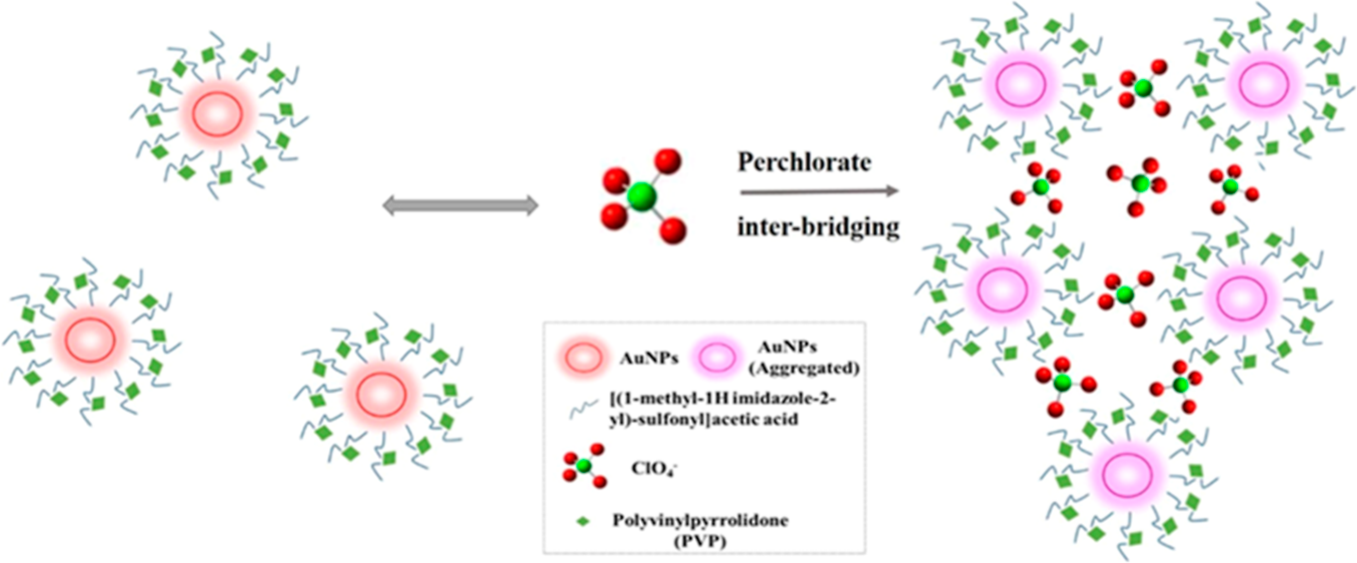

A rapid and convenient nanoparticle(NP)-based colorimetric sensor was developed for determining the propellant oxidant, ammonium perchlorate (AP). The sensing element was manufactured by modifying gold nanoparticles (AuNPs) with [(1-methyl-1*H*-imidazol-2-yl)sulfanyl]acetic acid, which is an imidazolium-based ionic liquid (IL), to produce the IL@AuNP nanosensor stabilized by polyvinylpyrrolidone. The used IL is an exceptional IL which can attach to AuNPs through the sulfanyl-S atom. The sensing principle was based on observing the red shift in the surface plasmon resonance band of AuNPs leading to NP aggregation as a result of anion−π interaction of perchlorate anion with the zwitterionic form of IL@AuNPs so as to bring opposite charges face-to-face, thereby reducing the overall surface charge of NPs. The surface plasmon resonance band of AuNPs at 540 nm shifted to 700 nm as a result of aggregation. The ratiometric sensing was performed by dividing the absorbance at 700 nm to the absorbance at 540 nm and correlating this ratio to the AP concentration. The limit of detection and limit of quantification of the sensor for AP were 1.50 and 4.95 μM, respectively. Possible interferences of other energetic substances and common soil ions in synthetic mixtures were also investigated to achieve acceptable recoveries of analyte. This work may pioneer similar sensing systems where the overall anionic charges of IL-functionalized AuNPs are exceptionally reduced by an analyte anion (perchlorate), thereby forcing NPs to aggregate.

## 1 Introduction

Ammonium perchlorate (AP) is an important oxidizer energetic material used in rocket propellants due to its high oxygen content. While energetic materials generally consist of carbon, oxygen, nitrogen, and hydrogen, the presence of chlorine surrounded by four oxygen atoms in a perfect tetrahedral environment in AP structure makes it different from other similar propellants.^1^ Most of the anthropogenic perchlorate is manufactured as AP with the purpose of oxidizing aluminum fuel in rockets and missiles. Its salts are also used in air bag inflators, nuclear reactors, lubricating oil additives, electroplating, tanning and leather finishing, painting, pyrotechnics, and munitions.^2^ Natural and man-made perchlorate may enter the food chain. Even Martian soil was shown to contain significant amounts of perchlorate, imparting a strongly oxidizing nature to soil.^3^ Thus, the development of sensitive and selective analytical methods for its determination constitutes an analytical challenge for building a safe society and a green environment.

For the quantitative determination of AP from propellant energetic materials, there are quite few colorimetric and nanomaterial-based analytical devices in the literature. Microfluidic paper-based portable analytical devices have been developed for the qualitative determination of explosives, such as propellant energetic materials for in-field analysis, on which perchlorate was detected with the methylene blue (MB) color reaction.^4^ Using the electrostatic interaction between cysteamine-modified gold nanoparticles (AuNPs) and the perchlorate anion, a surface enhanced Raman spectroscopy assay was designed.^5^ Keskin *et al.* have developed a colorimetric method based on the aggregation of cationic dye-modified AuNPs in the presence of AP.^6^ Although ion chromatography (IC) is one of the most widely used methods for the determination of perchlorate, interference effects of sulfate, oxalate, and other ions are a problem. For this reason, the AS16 column was used to separate perchlorate in the EPA standard method. A limit of detection (LOD) value of 0.53 μg/L was obtained using the conductivity detector.^7^ IC was applied by Sungur and Sangün in a wide variety of complex matrices such as soil, water, milk, fish, vegetables and fruits with LOD values between 0.24 and 0.59 μg/kg.^8^ For perchlorate determination, there are methodologies such as IC-tandem mass spectrometry,^9,10^ liquid chromatography-tandem mass spectrometry,^11,12^ and the ion selective electrode^13^ method also existing in the literature.

Noble metal nanoparticles (NM-NPs) are frequently used in the design of sensors, owing to their magnificent physical–chemical properties and surface plasmon resonance (SPR) absorption.^14,15^ The incident light can penetrate the metal and polarize the conduction electrons and the resulting oscillation of localized surface plasmons (LSPs) is distributed over the whole particle volume. Only light at the appropriate wavelength (known as the SPR absorption maximum or λ_max_) in resonance with the oscillation is able to excite the LSPs.^16^ Because this wavelength falls within the visible–NIR range, colorimetric sensors can be developed for specific analytes at the highest molar extinction coefficients for NM-NPs, envisaged by the Mie theory.^17^ The extrinsic size effect (*i.e.,* a retardation effect due to the excitation of multipolar plasmon modes when NP size increases with respect to the resonant electromagnetic wavelength) appears in the optical absorption spectrum as a broadening and bathochromic shift of the SPR band for increasing sizes of NM-NPs.^16^ The extraordinary properties of NM-NPs are highly dependent on particle size, being a function of the reducing agent that reacts with the metal salt.^18^ Among various noble metals, Au is more preferred in a NP form because it has an inert structure and easy surface modification. AuNPs cluster/aggregate depending on the interparticle distance.^14^ In 1857, Michael Faraday published the first work on the synthesis of AuNPs in a colloid.^19^ In recent years, nanosensors have been developed by modifying the surface of AuNPs with functional molecules.^20^ Also, various stabilizing agents, especially polymers such as polyvinylpyrrolidone (PVP), are widely used in the synthesis of AuNPs.^21^ PVP is a highly stable polymer with inert physicochemical properties over a wide pH range, containing a strong hydrophilic component (pyrrolidone moiety) and an important hydrophobic group (alkyl group).^22^ The PVP–Au interaction is based on electron pair exchange between the nitrogen or carbonyl oxygen of the PVP molecule and the hybrid orbitals of Au ions.^23^ Similar studies explaining this interaction exist in the literature.^24−26^

Ionic liquids are special salts in the liquid state, and the dissolution and stabilization of metal ions by ionic liquids (ILs) are superior compared to conventional organic compounds. Due to their low cost, easy synthesis, low vapor pressure, high lattice energy, and chemical/electrochemical stability, they have attracted great interest.^18^ The combination of ILs and NPs is very popular,^27^ involving the use of various IL derivatives with AuNPs.^18,20^

In the proposed study, it was aimed to develop a selective and easy-to-apply molecular spectroscopic sensor based on the aggregation of AuNPs in perchlorate determination. For manufacturing the NP-based perchlorate sensor, IL@AuNPs, AuNPs were synthesized and modified with NaBH_4_ (used as a reducing agent) and PVP (used as a stabilizing agent) in the presence of imidazolium cation-based IL. The IL in the cationic and zwitterionic form was attached to the AuNP surface by electrostatic interaction. At the working pH of 8, the zwitterionic IL is in reverse micelles form, and the less-hydrated perchlorate ion has the ability of incorporation, distributing into the interior of these reverse micelles^28^ so as to bring the IL@AuNPs closer together. When perchlorate anions inter-bridge the IL@AuNPs, aggregation occurs, enabling the indirect detection of ClO_4_^–^. The developed IL@AuNP sensor gives a SPR peak at 540 nm, and a new peak is formed at 700 nm as a result of NP aggregation when the sensor is applied to AP samples. Quantitative evaluation was done by taking the ratio *A*_700nm_/*A*_540nm_ (*i.e.,* by proportioning the absorbance at 700 nm to the absorbance at 540 nm) and correlating this ratio with the AP concentration to build a ratiometric sensor.

## 2 Methods

### 2.1 Chemicals

Sodium borohydride (NaBH_4_), [(1-methyl-1*H*-imidazol-2-yl)sulfanyl]acetic acid (IL), and PVP were purchased from Sigma-Aldrich (St. Louis, Missouri, USA). Tetrachloroauric(III) acid (HAuCl_4_, 99.99% pure-trace metal basis, 30% wt in dilute HCl) was purchased from Sigma-Aldrich (St. Louis, Missouri, USA). Disodium hydrogen phosphate dihydrate (Na_2_HPO_4_·2H_2_O) and sodium dihydrogen phosphate dihydrate (NaH_2_PO_4_·2H_2_O) were obtained from Sigma-Aldrich (St. Louis, Missouri, USA). AP was kindly donated by ROKETSAN Corporation (*i.e.*, an establishment of Turkish Armed Forces Foundation). The explosive materials 2,4,6-trinitrotoluene (TNT), 1,3,5,7-tetranitro-1,3,5,7-tetraazacyclooctane (HMX), 1,3,5-trinitro-1,3,5-triazacyclohexane (RDX), 2,4,6-trinitrophenyl-*N*-methylnitramine (tetryl), and pentaerythritoltetranitrate (PETN) were kindly supplied by the Mechanical and Chemical Industry Corporation (Makine Kimya Endustrisi Kurumu, MKEK) of Turkey from previous projects. Picric acid was purchased from Sigma-Aldrich.

### 2.2 Apparatus

AuNPs were synthesized and modified using an IKA C-MAG heater equipped with a magnetic stirrer. The spectra and absorption measurements were recorded in matched Hellma Suprasil quartz cuvettes using a Shimadzu UV-1800 UV–vis spectrophotometer (Kyoto, Japan), and the thickness of the optical cuvettes was 10 mm. Scanning transmission electron microscopy (STEM)–energy dispersive X-ray spectrometry (EDS) and measurements of NPs were performed using a Quattro FEG ESEM. Dynamic light scattering (DLS) measurements were carried out at a temperature of 25.0 ± 0.2 °C in water using a 90Plus particle size analyzer (Brookhaven Instruments, USA) equipped with a 35 mW HeNe laser. The infrared spectra were recorded by diffuse reflectance infrared Fourier-transform spectroscopy using an Alpha T model spectrophotometer (Bruker, Germany) in the 4000–400 cm^–1^ range. X-ray photoelectron spectroscopy (XPS) was conducted using a K-Alpha spectrometer (Thermo Fisher, USA) employing a monochromated Al Kα X-ray source (*hv* = 14,686.6 eV).

### 2.3 Preparation of Solutions

For synthesizing AuNPs, a 17% HAuCl_4_ solution was used as stock and diluted with water to freshly prepare 0.01% (v/v) auric acid working solution. A solution of 1.0 × 10^–2^ mol L^–1^ sodium borohydride (NaBH_4_) used as a reducing agent in AuNP synthesis was prepared daily. The IL solution used in the modification of AuNPs was prepared by diluting a suitable stock solution with H_2_O to be at 1.0 × 10^–4^ mol L^–1^. The phosphate buffer solution at pH = 8.0 [containing 0.5 mol L^–1^ sodium dihydrogen phosphate dihydrate (NaH_2_PO_4_·2H_2_O) and 0.5 mol L^–1^ disodium hydrogen phosphate dihydrate (Na_2_HPO_4_·2H_2_O)] was prepared in ultrapure water. A fresh solution of 1.0 × 10^–4^ mol L^–1^ NH_4_ClO_4_ was prepared from a stock solution and kept at +4 °C. AP at 10 μM was assayed in the presence of 1- and 5-fold concentrations of common ions, namely Cl^–^ (NaCl), NO_3_^–^ (NaNO_3_), SO_4_^2–^ (Na_2_SO_4_), NO_2_^–^ (NaNO_2_), CH_3_COO^–^ (NaCH_3_COO), HCO_3_^–^ (NaHCO_3_), B_4_O_7_^2–^ (Na_2_B_4_O_7_), F^–^ (NaF), NH_4_^+^ (NH_4_Cl), K^+^ (KNO_3_), Mg^2+^ [Mg(NO_3_)_2_], Ca^2+^ (CaCl_2_), Fe^3+^ (FeCl_3_), and Cu^2+^ (CuCl_2_). The preparation and dilution of all solutions were made with ultrapure water (H_2_O) throughout. Binary mixtures of common energetic materials, principally composed of RDX, TNT, HMX, tetryl, PETN, and picric acid, were prepared at 100 μM concentration (*i.e.*, 10-fold of the analyte) in acetonitrile/water (1:1, v/v) and AP was added at a final concentration of 10 μM.

### 2.4 Synthesis and Modification of AuNPs

In an Erlenmeyer, a mixture of 0.01% HAuCl_4_, 1 mL of 1.0 × 10^–4^ mol L^–1^ IL, and 25 mL of ultrapure water was stirred on a magnetic stirrer at 750 rpm for 10 min. Then, 2 mL of 1.0 × 10^–2^ mol L^–1^ NaBH_4_ solution was added dropwise and after stirring for 30 min with the addition of 0.1 g of PVP, the contents were left overnight for modification. The formation of IL-modified AuNPs (IL@AuNPs) was observed by the color change from pale yellow to claret red-purple. The absorption spectrum of the synthesized AuNP solution, as well as the absorbances at 540 and 700 nm, were recorded using a UV–vis spectrophotometer.

### 2.5 Spectrophotometric Application of AP Sensor

A volume of 0.3 mL of solvent (blank) or sample (AP) solution within 5.0–22.5 μM final concentration range was introduced to a test tube, and 0.5 mL of IL@AuNPs and 0.3 mL of 0.5 M pH 8 phosphate buffer solution were added to the tube. After 7 min of reaction time, the color changes from red to purple due to aggregation in the vial with increasing perchlorate concentrations were examined, and the absorption changes of each solution were measured against water at both 700 and 540 nm wavelengths.

### 2.6 Determination of AP in the Presence of Common Soil Ions

Analyte recovery values were found by preparing an AP solution with a final concentration of 10 μM, adding solutions of Cl^–^ (NaCl), NO_3_^–^ (NaNO_3_), SO_4_^2–^ (Na_2_SO_4_), NO_2_^–^ (NaNO_2_), CH_3_COO^–^ (NaCH_3_COO), HCO_3_^–^ (NaHCO_3_), B_4_O_7_^2–^ (Na_2_B_4_O_7_), F^–^ (NaF), NH_4_^+^ (NH_4_Cl), K^+^ (KNO_3_), Mg^2+^ [Mg(NO_3_)_2_], Ca^2+^ (CaCl_2_), Fe^3+^ (FeCl_3_), and Cu^2+^ (CuCl_2_) (at 1- to 5-fold mole ratio to AP) and then applying the proposed sensor.

In order to eliminate the interference effects of cations, Na_2_EDTA was used as a masking agent (metal–EDTA mole ratio 5:0.25 for Ca^2+^ and Mg^2+^) and then the proposed method was applied.

### 2.7 Determination of AP in Explosive Mixtures

Binary mixture solutions of RDX, TNT, HMX, tetryl, PETN, and picric acid (10-fold of AP, mole ratio) with 10 μM AP were prepared in acetonitrile/water (1:1 volume ratio). The developed sensor was applied to these mixtures to find the percentage recovery values of AP.

### 2.8 Extractive Separation of TNT from AP–TNT Binary Mixture

To 5 mL of AP–TNT binary mixture (1:10 mole ratio) in 1:1 acetonitrile/water were added 0.5 mL of 5% NaOH and 4 mL of 7.5 × 10^–3^ mol L^–1^ aqueous cetyl pyridinium bromide, and the mixture was extracted with 7.5 mL of isobutyl methyl ketone (IBMK). The colored Meisenheimer anion formed as a result of the reaction between TNT and NaOH formed an ion pair with cetyl pyridinium bromide, and TNT was extracted into the organic phase.^29^ AP remained in the aqueous phase and was filtered through a double-fold blue band filter paper. The percentage recovery values of AP with the recommended method were reported.

### 2.9 Determination of Perchlorate in a Real Sample and Validation of the Proposed Sensor against the Extraction Spectroscopic Procedure

For preparing the sparkler (*i.e.,* a firework that throws off brilliant sparks on burning) solution, a 0.6366 g sample was dissolved in water and diluted to 50 mL. The sample was dissolved homogeneously in an ultrasonic bath. After filtration through a filter paper (blue band) and dilution to a final volume of 50 mL, this clear solution was 20-fold diluted with H_2_O before detection of AP.

A volume of 5 mL of diluted sparkler filtrate, 1 mL of cetyl trimethyl ammonium bromide, and 5 mL of dichloromethane (DCM) were added to a shaker tube. After agitation of the mixture for 30 s and letting the separation of two phases, the organic phase was withdrawn and the solvent DCM evaporated. The residue was diluted to 50 mL by dissolving with ultrapure water. The sensing procedure was repeated on five repetitive samples identically prepared. The same solutions as samples were 10-fold diluted for spectrophotometric measurements using a pre-established extraction spectroscopic procedure^30^ for comparing the findings.

The pre-established extraction spectroscopic procedure was applied as described in the literature.^30^ A volume of 10 mL of AP solution (remaining within an initial concentration range of 4.0 × 10^–6^ to 5.0 × 10^–7^ mol L^–1^) was taken to a shaker tube, and 0.5 mL of 0.1 N sulfuric acid, 1 mL of 4.0 × 10^–4^ mol L^–1^ MB, and 10 mL DCM were added. The mixture was shaken for 30 s and allowed for the separation of the two phases. The electrostatic complex comprised of MB cation and perchlorate anion (*i.e.,* large cation–large anion pair having a weak hydration sphere) remained in the organic phase, which was withdrawn with a pipette for spectrophotometric measurement against a reagent blank at 655 nm wavelength. The calibration curves were drawn between the concentration of AP and organic phase absorbance. Five repetitive sparkler samples were used for AP determination and the results found with the recommended and reference method were statistically compared.

### 2.10 Statistical Analysis

Descriptive statistical analyses were performed using Excel software (Microsoft Office 2013) for calculating the means and the standard error of the mean. Results were expressed as the mean ± standard deviation (SD). Statistical comparison of the proposed method of AP determination against the extraction spectroscopic procedure existing in the literature^30^ was made by means of Student’s *t*- and *F*-tests for evaluating accuracy and precision, respectively.

## 3 Results and Discussion

### 3.1 Proposed Detection Mechanism for Perchlorate

Anion−π interactions can be thermodynamically favorable and show a great potential in the near future for the design of selective anion receptors or scaffolds to build supramolecular architectures that may lead to colorimetric sensors.^31^ Anion−π supramolecular interaction may occur between electron-deficient aromatic π-systems and an anion, principally consisting of electrostatic forces and ion-induced polarization. In case when self-assembled monolayers are attached to surfaces, amplification of sensory response at reduced water solubility is possible.^32^ As a part of noncovalent interactions, anion−π interactions may motivate diverse supramolecular processes including anion recognition and selective sensing, anion-directed self-assembly of sophisticated architectures, and agglomeration/aggregation. In the manufacture of sensors, anion−π interactions may act synergistically with other noncovalent bindings and hydrogen bonding.^33^

In our case, anion−π interaction occurs between ClO_4_^–^ anion and imidazolium cation of zwitterionic IL (Im–COOH: Im^+^COO^–^) attached on the AuNP surface (IL@AuNPs).

Zwitterionic surfactants form a special class of dipolar organic compounds which can form normal and reverse micelles without the need for a cosurfactant. Dipolar ionic surfactants can form normal and reverse micelles of zwitterionic character, which can stabilize NPs in water and organic media.^28^ In the presence of the rather hydrophobic perchlorate ion of tetrahedral geometry, these micelles can become anionoid by incorporating ClO_4_^–^ on the micellar surface. The free perchlorate ion, being less hydrated than other similar anions, shows a stronger interaction with the imidazolium moiety of the surfactant and can be distributed in the interior of the reversed micelle.^28^ These micellar aggregates can form more easily on AuNPs. Perchlorate anions facilitate the formation of a zigzag 2-D network structure assembly with imidazolium ions.^34^ Here, we have shown that when perchlorate anions inter-bridge the IL@AuNPs, aggregation occurs, enabling the detection of ClO_4_^–^. A regional decrease in the polarity of the medium facilitated by an asymmetric distribution of the charges (*e.g.*, as a result of dipole–dipole interaction) on NPs also enhances NP enlargement accompanying aggregation.^35^

**Scheme 1:**
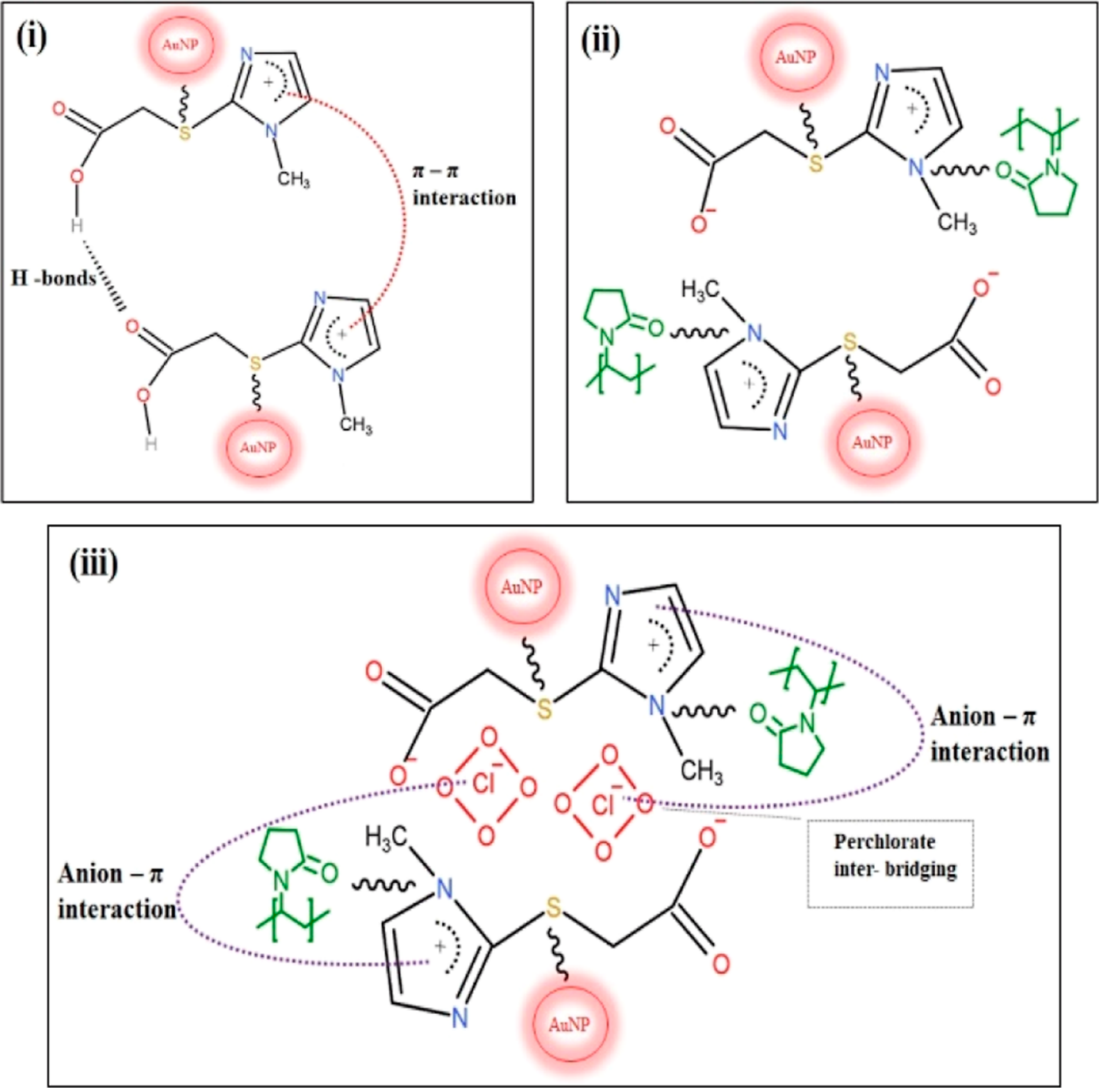
Proposed Mechanism for IL@AuNP Aggregation Detection of ClO_4_^–^

Thus, in our case, the proposed perchlorate probing mechanism consists of three stages, as schematized in Scheme 1:

(i) When IL@AuNPs are prepared at acidic pH (3–4), self-aggregation occurs because of the H-bonding interactions between the −COOH groups on NPs. When these NPs are coated with PVP, H-bonding interactions are weakened.
(ii) At the optimal pH of 8, the zwitterionic structure is disrupted, and the conjugate base (acetate) prevails. The IL@AuNPs become an ideal probe for perchlorate because there is a net negative charge on NPs (arising from the carboxylate-rich groups) preventing aggregation.
(iii) When the weakly-hydrated analyte (perchlorate ion) meets the probe, it inter-bridges NPs bringing the negative (acetate) and positive (imidazolium) parts of the zwitterionic surfactant (attached on separate NPs) together face-to-face. Thus, the reduction of the surface charge of NPs brings about aggregation, enabling the selective detection of ClO_4_^–^.

### 3.2 Characterization of IL@AuNPs

**Figure 1:**
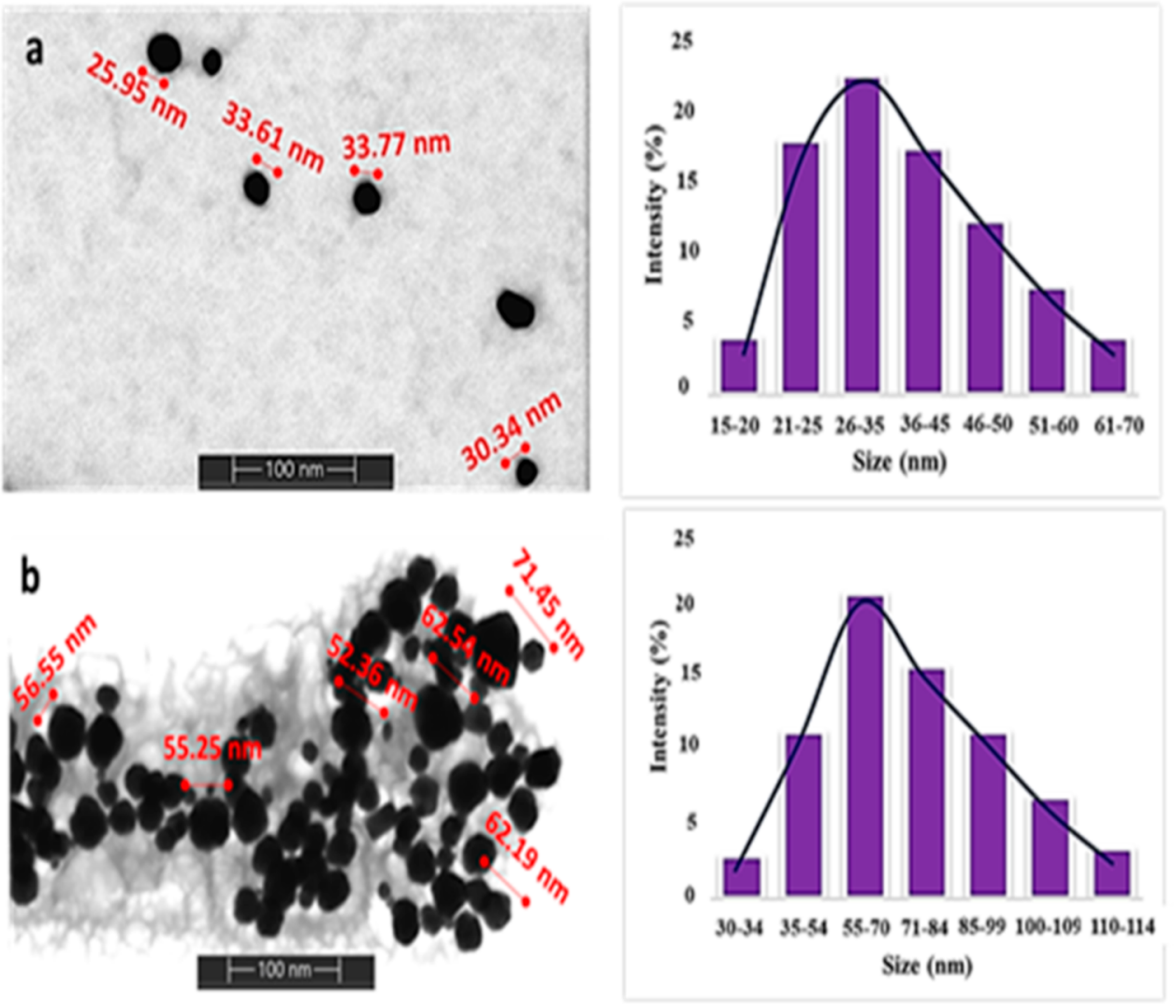
STEM images and DLS histograms of IL@AuNPs in the absence of AP (a) and in the presence of AP (b) {[AP]: 22.5 μM}.

STEM images and size distribution histograms (DLS) of IL@AuNP samples were investigated in the presence and absence of AP. When the STEM image and DLS histogram results of IL@AuNPs were examined, it was seen that the average particle size is in the range of 25–35 nm (Figure 1a). With the addition of AP to the colloidal solution, the average particle size increases to 55–70 nm as a result of the formation of aggregates composed of IL@AuNPs + AP (Figure 1b).

**Figure 2:**
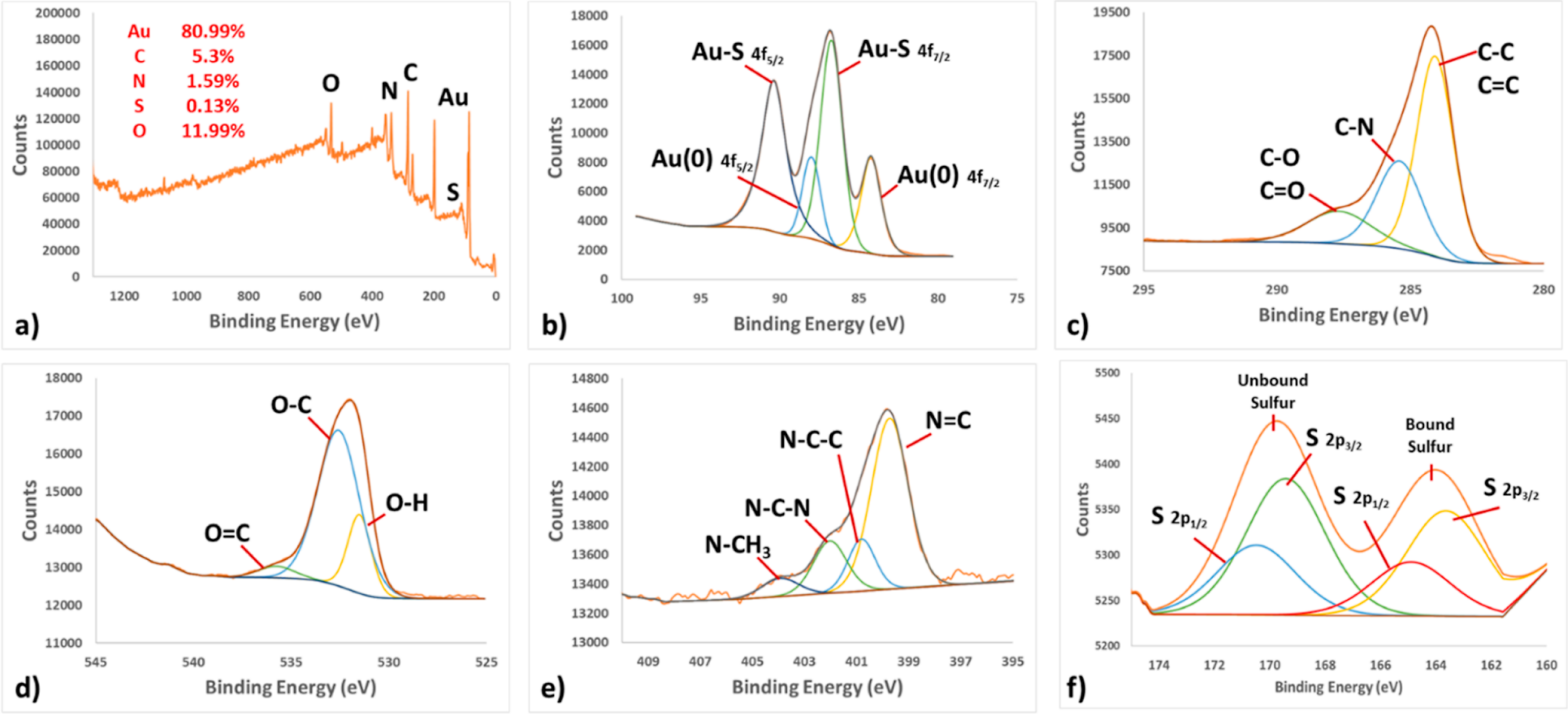
(a) XPS survey spectra of IL@AuNPs. XPS spectra of (b) Au, (c) C, (d) O, (e) N, and (f) S atoms.

**Figure 3:**
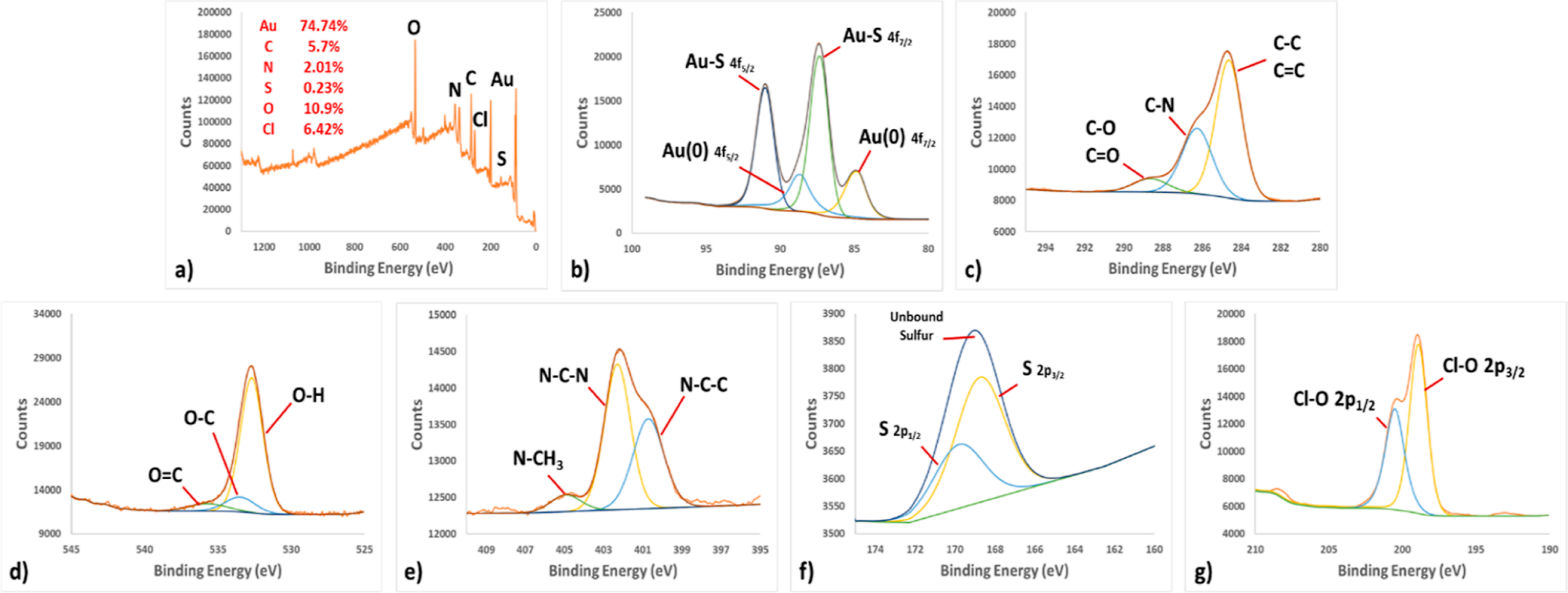
(a) XPS survey spectra of IL@AuNPs/AP. XPS spectra of (b) Au, (c) C, (d) O, (e) N, (f) S, and (g) Cl atoms.

**Figure 4:**
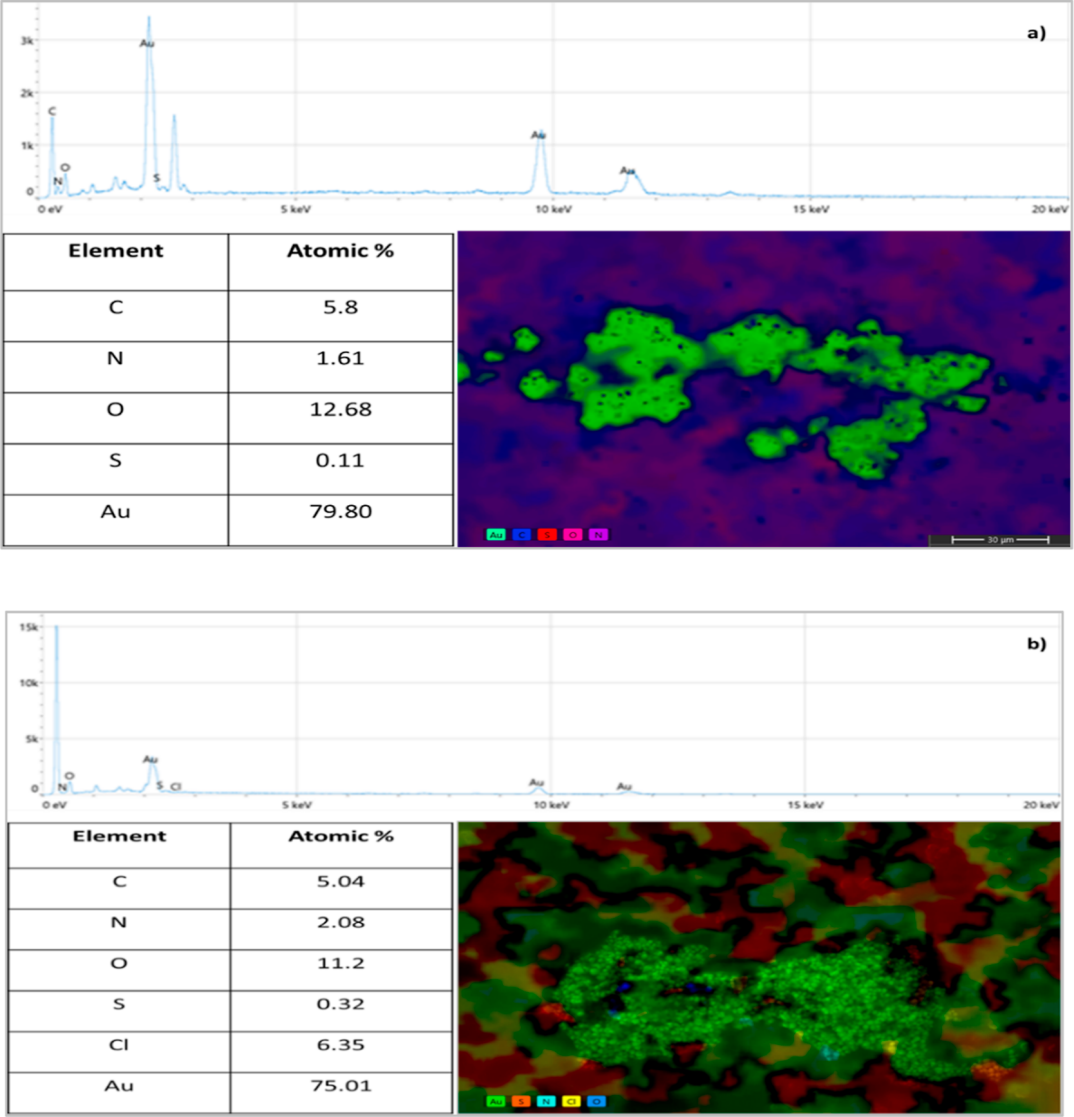
EDS spectra, color STEM images, and percentage atomic ratios of IL@AuNPs in the absence of AP (a) and in the presence of AP (b).

XPS technique was applied in order to better understand how AuNPs are modified with IL and also how modified AuNPs interact with perchlorate anion. Figure 2a shows the XPS survey scan of modified AuNPs. When the spectrum is examined, the Au (80.99%) of the NP and the C (5.3%), N (1.59%), S (0.13%), and O (11.99%) peaks of the IL as the modifying agent are clearly seen. Figure 2b shows the detailed XPS spectrum of Au. Au (O) (88.18 and 84.08 eV) and Au–S (90.28 and 86.58 eV) peaks in the spectrum confirm that the IL is bound to the AuNP *via* the sulfur atom. On the other hand, C–O/C=O (287.78 eV), C–N (285.38 eV), and C–C/C=C (283.90 eV) peaks supporting the structure of the IL are seen in the C XPS spectrum (Figure 2c). In the O spectrum, O=C (535.88 eV), O–C (532.88 eV), and O–H (531.28 eV), which are the peaks belonging to the carboxylic acid group of the IL, are seen (Figure 2d). When Figure 2e is examined in detail, there are N–CH_3_ (403.88 eV), N–C–N (402.18 eV), N–C–C (400.68 eV), and N=C (399.48 eV), which are N peaks in the structure of IL having different chemical environments. Finally, when Figure 2f is examined, four peaks belonging to two different binding regions of the sulfur atom are seen. The first of these regions is the unbound sulfur, and the other is the bonding sulfur. In the unbound sulfur region are the S 2p_1/2_ and S 2p_3/2_ peaks of oxidized sulfur at 170.28 and 169.48 eV, respectively. On the other hand, in the bonding sulfur region, there are S 2p_1/2_ and S 2p_3/2_ peaks of sulfanyl sulfur that interacted with Au at 164.78 and 163.58 eV, respectively.^36^ All these spectra show that the AuNPs were successfully modified with the IL. XPS survey spectrum of modified AuNPs after interaction with perchlorate is given in Figure 3a. In addition to the survey spectrum of the modified AuNPs, the Cl (6.42%) peak is observed in the spectrum. The Au, C, and O spectra in Figure 3b–d are similar to the modified AuNP (Figure 2b–d). In Figure 3f, the S 2p_1/2_ and S 2p_3/2_ peaks of sulfur are seen at 169.98 and 168.78 eV, respectively. These peaks are attributable to the oxidized sulfur.^37^ In the developed method, it was suggested that perchlorate interacts with the π-bonds in the ring structure of the IL and thus AuNPs are aggregated. In accordance with this suggestion, when the N spectrum is examined, it is seen that the N=C peak disappears and the N–C–N (402.18 eV) and N–C–C (400.68 eV) peaks are intensified. This confirms that perchlorate interacts with the ring π-bonds of the IL. Finally, in Figure 3g, there is the spectrum of the Cl atom. On the other hand, Cl–O (200.38 and 198.98 eV) bond peaks are clearly seen in the spectrum, indicating the presence of perchlorate in the medium. STEM–EDS images of IL@AuNP samples were investigated in the presence and absence of AP. Figure 4 shows the color STEM images, EDS spectra, and percent atomic ratios in the sample of IL@AuNPs in the absence (Figure 4a) and presence (Figure 4b) of AP. STEM–EDS results obtained are compatible with XPS analysis results. When the spectra in Figure 4a,b are examined, it can be seen that the samples do not contain any impurities.

The interaction between imidazolium-based IL-modified AuNPs and AP was investigated by measuring the ζ potential of IL@AuNPs with and without AP. IL@AuNPs show a ζ potential value at (−) 22.8 mV at pH = 8, whereas this value drops to (−) 3.07 mV after contact of AP with IL@AuNPs. ζ potential measurements show that at the working pH of 8, the zwitterionic structure of imidazolium-based IL-modified AuNPs is disrupted and the conjugate base (acetate) dominates, leaving a distinct negative charge (−22.8 mV) on NPs. In similar ILs without a sulfanyl group, the highest p*K*_a_ value attributed to the imidazole group is ≥7 and the lowest one to the carboxylate group is around 3 at 25 °C.^38^ This means that the H-bonding possibility bringing about aggregation in Scheme 1(i) is not possible at the working pH of 8 because the acetate groups of the IL are deprotonated, as shown in Scheme 1(ii). Thus, IL@AuNPs are perfectly stable having a distinct negative charge at the working pH. When the perchlorate ion meets IL@AuNPs, it inter-bridges NPs bringing the oppositely charged (negative acetate and positive imidazolium) parts of the zwitterionic liquid symmetrically together. Thus, charge neutralization occurs by decreasing the surface charge (*i.e.,* down to −3.07 mV) initiating aggregation.

The infrared spectra (KBr pellet) of pure AP (a), [(1-methyl-1*H*-imidazol-2-yl)sulfanyl]acetic acid (IL) (modifying agent) (b), pure PVP (c), imidazolium-based IL-modified AuNPs (d), and IL@AuNPs interacted with AP (e) were taken. The infrared spectra (KBr pellet) are provided in Figure S1 of Supporting Information.

### 3.3 Optimization Parameters of the Sensor

In the determination of AP with the proposed sensor, the medium pH and the buffer solution used to keep it constant are very important parameters. In acidic medium, the nitrogen atom of IL [(1-methyl-1*H*-imidazol-2-yl)sulfanyl]acetic acid structure is protonated. Under conditions when the zwitterionic form of IL on AuNPs prevail, the NPs may still stay apart in the absence of an inter-bridging agent such as perchlorate which may penetrate the reverse micelles (Scheme 1(ii)). In weak alkaline pH medium, perchlorate ions may be incorporated in supramolecular architectures by distributing into the reverse micelle form of IL@AuNPs, bringing them together and aggregation occurs (Scheme 1(iii)). For creating a weak alkaline medium, tris and phosphate buffers at alkaline pH were used. The tris buffer (p*K*_a_ = 8.1) is half-protonated at the working pH of 8, at which the IL is weakly protonated. Thus, the tris buffer ammonium groups compete with the cyclic ammonium of IL for the perchlorate anion, and an aggregation-based perchlorate sensor does not operate, whereas a phosphate buffer at the same pH is not a competitor. Considering these, a mixture of Na_2_HPO_4_–NaH_2_PO_4_ was chosen as the optimal buffer solution for adjusting the pH of sensing medium to pH = 8.

In order to determine the optimal reaction time, the sensor response to the AP solutions was examined for half an hour, and it was observed that the sample solution color shifted to purple after the first 7 min. Therefore, absorbance measurements were made after 7 min.

### 3.4 Analytical Performance of the Sensor

The developed IL@AuNP sensor shows a characteristic LSPR band at 540 nm. When the proposed method was applied for AP at different concentrations, a change from red to purple was observed in the color scale of the solutions. Accordingly, a new peak formation was observed in the range of 600–800 nm in the absorption spectrum. The maximal absorbance value of these peaks (as a result of NP aggregation) cannot be observed at a fixed wavelength. For this reason, absorbance values at 700 nm were taken as the basis to represent the spectral change in SPR absorption of aggregated NPs. The absorbance ratio (*A*_700nm_/*A*_540nm_) was selected for quantitative evaluation because the absorbance at 700 nm steadily increased, whereas those at 540 nm slightly decreased with increasing concentrations of AP. Therefore, these peaks were proportioned to each other, and the absorbance ratio of the reference solution was subtracted from that of the sample solution to obtain a corrected absorbance of the ratiometric sensor.

Corrected absorbance values [(*A*_700nm/540nm_)_sample_ – (*A*_700nm/540nm_)_blank_] were calculated by using absorbance values at 700 and 540 nm for AP samples. A calibration curve was created for the quantitative analysis of perchlorate with the corrected absorbance values obtained, and a good linear curve was obtained within the final concentration range of 5.0–22.5 μM of AP.
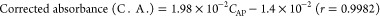
where *C*_AP_ is the AP concentration (in μM) in final solution.

**Figure 5:**
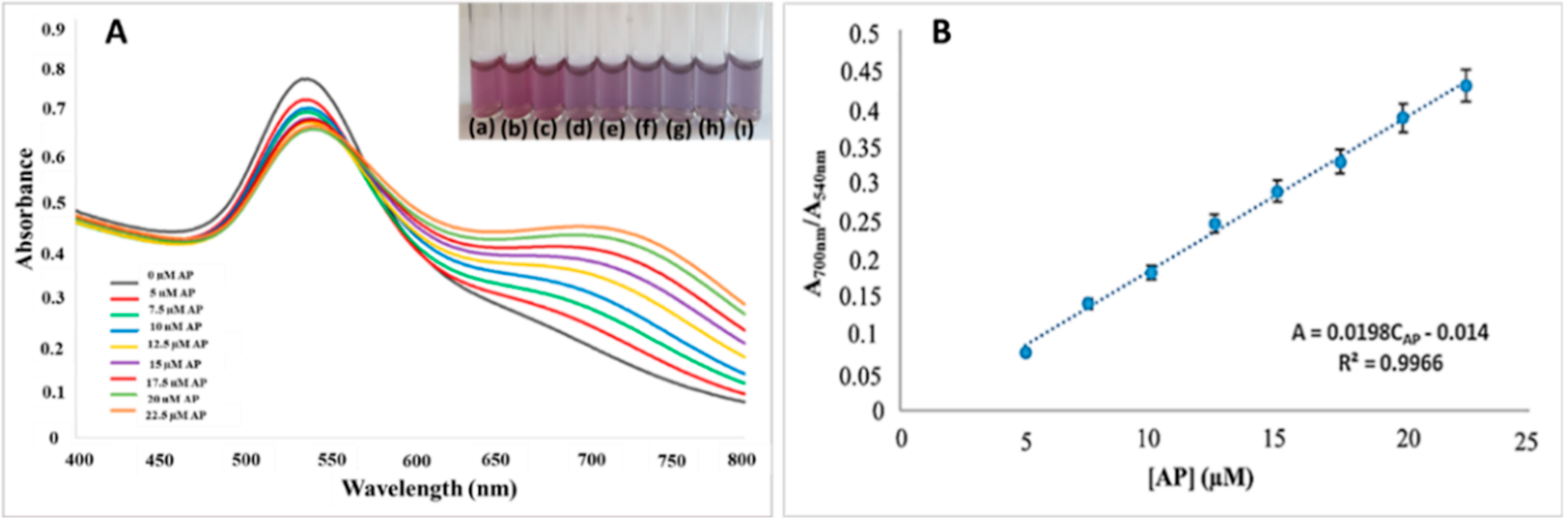
(A) Spectra of aggregated NPs in the visible range, using the recommended sensor for AP solutions at different concentrations and (inset) color changes of the test tubes containing (a) reference solution, (b) 5.0, (c) 7.5, (d) 10.0, (e) 12.5, (f) 15, (g) 17.5, (h) 20.0, (i) 22.5 μM AP. (B) Plot of the relative absorbance ratios of the IL@AuNP system against AP concentration.

The spectra and colors obtained by applying the proposed method to different concentrations of AP are shown in Figure 5A, and the calibration curve constructed with the corrected absorbance values obtained (*i.e.,* the ratio of absorbance recorded at 700 nm to that at 540 nm) is shown in Figure 5B.

The analytical performance parameters of the developed sensor were as follows:

Limit of detection (LOD): 1.50 μM and limit of quantification (LOQ): 4.95 μM.

The LOD was defined to be 3σ_bl_/*m* and LOQ equal to 10σ_bl_/*m*, where σ_bl_ denotes the SD of a blank and *m* is the slope of the calibration curve.

Five replicate measurements (*N* = 5) were performed to determine intra-assay and inter-assay precision, where the relative SDs (RSD %) for AP were found to be 5.97 and 14.75%, respectively. In addition, a comparison between this method and AuNP-based methods published in the literature for perchlorate determination with respect to the linear ranges and detection limits is listed in Table S1.

### 3.5 Investigation of Possible Interferences Caused by Common Soil Ions

An AP solution with a final concentration of 10 μM was examined in the presence of 1- and 5-fold common soil ions (Cl^–^, NO_3_^–^, SO_4_^2–^, NO_2_^–^, CH_3_COO^–^, HCO_3_^–^, B_4_O_7_^2–^, F^–^, NH_4_^+^, K^+^, Mg^2+^, Ca^2+^, Fe^3+^, and Cu^2+^) by implementing the developed sensor, and the recovery values of AP were calculated to obtain satisfactory results. The percentage recovery values of AP in the presence of common soil ions are shown in Table 1. For certain excessive amounts of interferent ions, EDTA was used to eliminate the interference effect, and the analyte recoveries observed in the presence of EDTA are also shown in Table 1 for three ions (Ca^2+^, Mg^2+^, and NO_2_^–^).

**Table 1:** Recoveries from 10 μM AP Solution Containing Potentially Interferent Common Soil Ions at a 1- to 5-Fold Concentrations

interferent ion	interferent ion final concentration (μM)	(analyte/interferent ion) mole ratio	recovery (%)	(analyte/cation–anion/EDTA) mole ratio	recovery (%)
Cl^–^	10	1	110		
	50	5	95		
NO_3_^–^	10	1	100		
	50	5	100		
SO_4_^2–^	10	1	110		
	50	5	90		
CH_3_COO^–^	10	1	101		
	50	5	102		
HCO_3_^–^	10	1	98		
	50	5	93		
B_4_O_7_^2–^	10	1	96		
	50	5	90		
F^–^	10	1	108		
	50	5	96		
NO_2_^–^	10	1	110	1:5:1	106
	50	5	126		
K^+^	10	1	95		
	50	5	90		
NH_4_^+^	10	1	101		
	50	5	109		
Mg^2+^	10	1	93	1:5:0.25	100
	50	5	85		
	100	10	106		
Ca^2+^	10	1	90	1:5:0.25	90
	50	5	83		
	100	10	102		
Fe^3+^	10	1	105		
	50	5	97		
Cu^2+^	10	1	110		
	50	5	87		

When the effect of NO_2_^–^ anion on the proposed method was investigated, NO_2_^–^ was found to interfere at 5-fold concentrations relative to AP (Table 1). NaNO_2_ behaves in a peculiar manner to have a high adsorption free energy at aqueous interfaces, where adsorption occurs as single ion-paired species rather than two translationally independent surface ions (*i.e.,* Na^+^ and NO_2_^–^). The interfacial stabilization of the ion pair in sodium nitrite (at concentrations as low as several millimolar levels) may cause it to be preferentially adsorbed relative to independently solvated ions^39^ and cause positive error. Another hydrated anion, EDTA (H_2_Y^2–^) could counteract this effect by displacing NaNO_2_ and allowing the ClO_4_^–^ ion to interact, thereby removing the interference effect of NO_2_^–^.

On the other hand, Ca^2+^ and Mg^2+^ ions caused a weak negative error in analyte recovery at 5-fold concentrations, which could be overcome by adding substoichiometric amounts of EDTA as a chelation agent for these alkaline earth cations.

It is also important to examine the role of ammonium ions in addition to the perchlorate ions of AP used in the method. According to the hydrophobicity order of anions reported as early as 1888 by Franz Hofmeister in his research work on serum albumin proteins, ClO_4_^–^ is the weakest hydrated anion producing the least salting-out effect on proteins, making perchlorate an imperfectly hydrated “chaotropic” anion. In other words, chaotropic anions cannot organize H_2_O molecules around them and consequently have a rather salting-in effect on proteins.^40^ Perchlorate anion may only weakly coordinate H_2_O molecules, having an average hydration number varying between 2.6 and 3.0.^41^ On the other hand, ammonium cation (NH_4_^+^) has an average hydration number of ≥8.^42^ Thus, the free perchlorate ion, being less hydrated than other similar anions, shows a stronger interaction with the imidazolium moiety of the surfactant and can be distributed in the interior of the reversed micelle bringing about aggregation of NPs, whereas the better hydrated ammonium cation may not show such an ability. The potential interference of ammonium on the proposed sensing system was investigated, and ammonium was shown not to interfere at 5-fold concentrations (relative to that of the analyte, perchlorate).

### 3.6 Determination of AP in Explosive Mixtures

**Figure 6:**
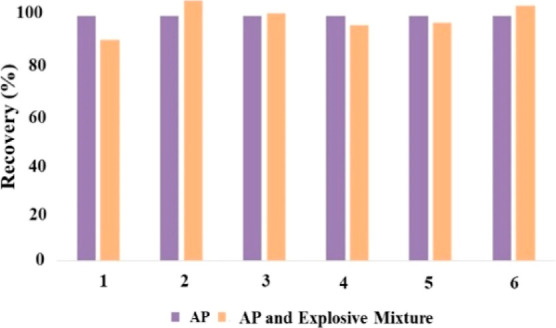
Recovery of AP in binary mixtures with other explosives (1:10 mole ratio of analyte/explosive). The explosives are 1: RDX, 2: TNT, 3: HMX, 4: tetryl, 5: PETN, and 6: picric acid (the purple column represents AP, the orange column represents binary explosive mixtures).

The widely known explosives such as TNT, RDX, HMX, tetryl, picric acid, and PETN were used in binary explosive mixtures together with AP. The developed method was applied to mixtures of AP with 10-fold (mol/mol) RDX, HMX, tetryl, picric acid, and PETN. The tested explosives at 10-fold levels did not affect the developed AP determination method in binary mixtures, where analyte recoveries were found between 90 and 104%. TNT showed interference at 10-fold concentration (by mole) of AP. At the working pH of 8, TNT partly forms a Meisenheimer anion, preventing perchlorate from entering the micelles. To eliminate this interference, the TNT Meisenheimer anion was extracted with CP^+^Br^–^ from alkaline aqueous solution into IBMK,^29^ and a 106% AP recovery was found. The recoveries of AP from binary explosive mixtures are shown in Figure 6.

### 3.7 Determination of AP with Extraction Spectroscopic Procedure and Method Validation in a Real Sample

The proposed sensor was applied for the determination of AP in sparkler samples and the validation of the proposed sensor was made with the pre-established extraction spectroscopic procedure^30^ in the literature. AP calibration solutions within 5 × 10^–7^ to 4 × 10^–6^ mol L^–1^ concentrations were analyzed with the literature extraction spectroscopic procedure and the calibration equation between absorbance (*A*) and concentration was *A* = 9.03 × 10^4^*C*_AP_ + 4.1 × 10^–1^ (*r* = 0.99) where *C*_AP_ is the concentration of AP in the final solution (mol L^–1^).

The proposed method for AP was compared with the reference method using 1.0 × 10^–5^ mol L^–1^ solutions (*N* = 5). For measurements with the extraction spectroscopic procedure, 1.0 × 10^–6^ mol L^–1^ AP solutions were used and then the results multiplied by the dilution factor. In accordance with the finding with the literature method (as 27.35%) used for comparison, the perchlorate content of the sparkler tested with the proposed sensing method was found to be 27.92%. Both methods showed no significant differences between the results (Table 2). Therefore, the proposed method was validated against the extraction spectroscopic procedure, and the *t*- and *F*-tests were used for comparing the population means and variances, respectively. The confidence level used in validation of AP findings was 95% for both *t*- and *F*-tests.

**Table 2:** Statistical Comparison of the Proposed Method with the Pre-established Extraction Spectroscopic Procedure for the Determination of AP in Sparkler Samples

	proposed sensor	extraction spectroscopic
mean concentration (w/w %)	27.92	27.35
SD (σ)	1.23 × 10^–2^	5.92 × 10^–1^
*S*^a^^,^^b^	9.65 × 10^–1^	
*t*^a^^,^^b^	1.67	
*t*_table_^b^	2.306	
*F*^b^	4.32	
*F*_table_^b^	6.39	

a *S*^2^ = {(*n*_1_ – 1)*s*_1_^2^ + (*n*_2_ – 1)*s*_2_^2^}/(*n*_1_ + *n*_2_ – 2) and *t* = (*a̅*_1_ – *a̅*_2_)/(*S*(1/*n*_1_ + 1/*n*_2_)^1/2^) where *S* is the pooled SD, *s*_1_ and *s*_2_ are the SDs of the two populations with sample sizes of *n*_1_ and *n*_2_ and sample means of *a̅*_1_ and *a̅*_2_ respectively [*t* has (*n*_1_ + *n*_2_ – 2) degrees of freedom]; and here, *n*_1_ = *n*_2_ = 5.

b Statistical comparison made on paired data produced with proposed and reference methods. The results given only on the row of the reference method.

## 4 Conclusions

There is great technological interest in AP due to its extensive use in solid propellant rockets. In the defense industry, there is a need to develop analytical methods in order to detect trace amounts of explosive substances and to carry out their determination with high sensitivity, speed, and accuracy. Based on these requirements, in this study, a simple and sensitive colorimetric assay method was developed for the determination of AP using IL@AuNPs. A zwitterionic imidazolium-based IL (having a sulfanyl group) was attached to the AuNP surface by the affinity of the sulfanyl-S atom for Au and electrostatic interaction to produce a nanosensor. In alkaline medium, the zwitterionic IL is in reverse micellar form and the perchlorate ion diffuses into these reverse micelles, bringing the IL@AuNPs together, forming a zigzag 2-D network structure. The perchlorate ion assembles the opposite charges of the zwitterionic surfactant that are bound to individual IL@AuNPs in the zigzag 2-D network structure, leading to charge neutralization and binding NPs together. Because the perchlorate anions bridge the IL@AuNPs, partial agglomeration occurs, enabling the detection of ClO_4_^–^. The presence of AP in the concentration range of 5.0–22.5 μM can be measured through the correlation between the absorbance ratio (*A*_700_/*A*_540_) and the AP concentration. As selective and sensitive AP determination methods in the literature are rare, it is thought that the developed method will fill an important gap. The detection limit of the developed AP determination method is as low as 1.50 μM. It is believed that the proposed detection method will find use in a diverse range of applications, such as crime scene investigation, on-site analysis, and decontamination of former military sites. This work is expected to pioneer similar sensing systems where the overall negative charges of IL-functionalized AuNPs are exceptionally reduced by a negatively charged analyte (perchlorate), thereby forcing the NPs to aggregate.

## Supporting Information Available

Infrared spectra (KBr pellet) of AP (pure), PVP (pure), [(1-methyl-1*H*-imidazol-2-yl)sulfanyl]acetic acid (IL) used as a modifying agent, imidazolium-based IL-modified AuNPs, and IL@AuNPs that interacted with AP and comparison table between the proposed method and AuNP-based methods published in the literature for perchlorate determination with respect to the linear ranges and detection limits presented is this paper (PDF)
