# Supplementary material for: Ionic Liquid-Modified Gold Nanoparticle-Based Colorimetric Sensor for Perchlorate Detection via Anion−π Interaction
Source: ACS Omega. 2022 Aug 3;7(32):28065–75. doi: 10.1021/acsomega.2c02078 (PMC9386817; doi:10.1021/acsomega.2c02078)
Supplement: Supplementary file 1 — ao2c02078_si_001.pdf [file ao2c02078_si_001.pdf]

SUPPORTING INFORMATION

Ionic Liquid Modified Gold Nanoparticles−Based Colorimetric Sensor for Perchlorate Detection *via* Anion-π Interaction

Büşra Keskin[†,‡], Ayşem Üzer[‡,*], Reşat Apak[‡,Ψ,**]

[†]Institute of Graduate Studies, Istanbul University-Cerrahpaşa, 34320 Avcilar, Istanbul, Turkey.

[‡]Department of Chemistry, Faculty of Engineering, Istanbul University-Cerrahpaşa, 34320 Avcilar, Istanbul, Turkey.

[Ψ]Turkish Academy of Sciences (TUBA), Bayraktar Neighborhood, Vedat Dalokay St. No:112, Çankaya, 06690 Ankara, Turkey.

*Corresponding Author; Prof. Dr. Ayşem Üzer (E-mail: auzer@iuc.edu.tr)

**Co-corresponding Author; Prof. Dr. Reşat Apak (E-mail: rapak@istanbul.edu.tr)

Supporting Information Contains

Abbreviations; The infrared spectra (KBr pellet) of AP (pure), PVP (pure), [(1-methyl-1H imidazol-2-yl)sulfanyl] acetic acid (ionic liquid, IL) used as a modifying agent, imidazolium-based ionic liquid−modified AuNPs, and IL@AuNPs interacted with AP (Figure S1); Comparison table between the proposed method and AuNPs-based methods published in the literature for perchlorate determination with respect to the linear ranges and detection limits (Table S1); References.

Abbreviations

AP, Ammonium perchlorate; PVP, Polyvinylpyrrolidone; IL@AuNPs, Ionic liquid modified gold nanoparticles; SERS, Surface enhanced Raman spectroscopy.

Supplementary Figures

The infrared spectra (KBr pellet) of pure AP (a), [(1-methyl-1H imidazol-2-yl)sulfanyl] acetic acid (ionic liquid, IL) (modifying agent) (b), pure PVP c), imidazolium-based ionic liquid−modified AuNPs (d) and IL@AuNPs interacted with AP (e) were taken. The spectrum in Figure S1a shows that although the perchlorate in the structure of ammonium perchlorate gives a peak in the fingerprint region, there are no sharp peaks in the spectrum. The N-H peak of ammonium is observed around 1573 cm^{-1}. In Figure S1b, characteristic peaks of the imidazolium-based ionic liquid used to modify the gold nanoparticles are clearly seen. The C=O stretching peak at 1645 cm^{-1}, the C=C stretching peak at 1553 cm^{-1} ,the C=N stretching peak at 1426 cm^{-1}, the C-N stretching peak at 1328 cm^{-1}, the C-O stretching peak at 1148 cm^{-1} and the CH_2 bending double peaks at ~948 cm^{-1} and ~874 cm^{-1} are characteristic peaks of imidazolium-based ionic liquid. In Figure S1c, the characteristic peaks of polyvinylpyrrolidone (PVP), which is attached to the surface of nanoparticles as a stabilizer, are clearly seen. The C=O stretching peak at 1566 cm^{-1}, the C=N stretching peak at 1383 cm^{-1} and the CH_2 bending

double peaks at ~938 cm^{-1} and ~877 cm^{-1} belong to the groups in the pyrrolidone ring of polyvinyl pyrrolidone. It is seen that the intensity of the C=O stretching peak (~1620 cm^{-1}) and C=C stretching peak (~1550 cm^{-1}) of the free IL@AuNPs (in the presence of PVP) (Figure S1d) decreases as a result of the interaction of the IL@AuNPs with AP (Figure S1e), giving rise to agglomeration of nanoparticles where the functional groups remain between the clusters. Similarly, the -CH_2 bending peaks (~930 cm^{-1} and ~880 cm^{-1}) seen in Figure S1d show an intensity decrease as a result of the interaction of IL@AuNPs with AP (Figure S1e). The decrease in the intensity of this peaks after the interaction of perchlorate with IL@AuNPs confirms our proposed aggregation mechanism. Also, a characteristic fellow peak of around 2500 cm^{-1} is observed in all spectra. Those peaks are the adsorbed CO_2 peak in the air.

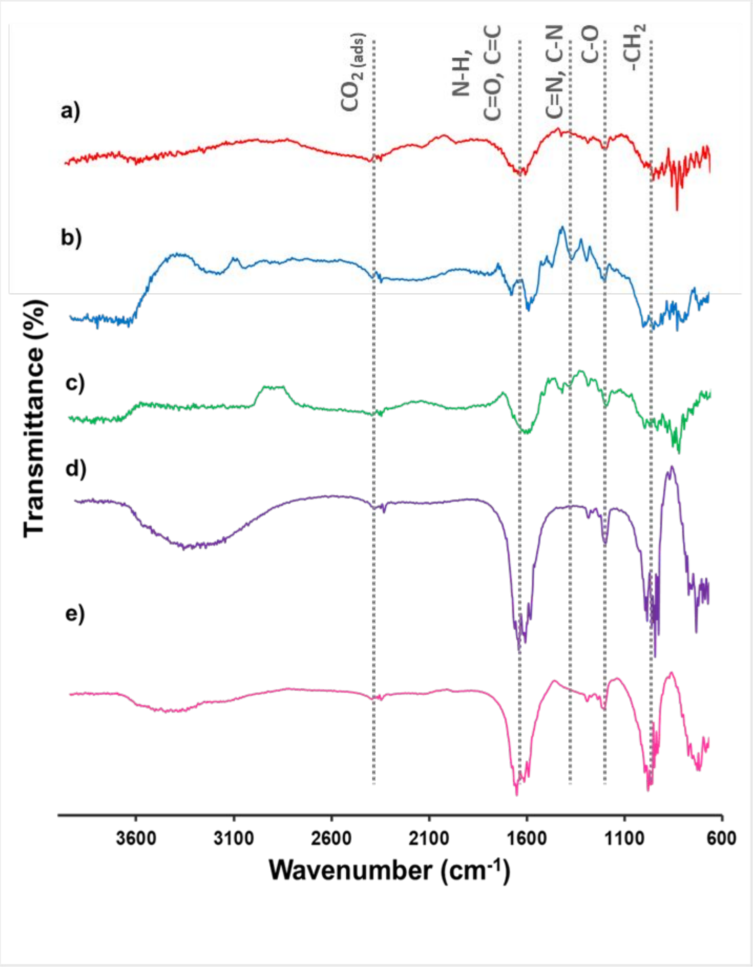

Figure S1. The infrared spectra (KBr pellet) of a) AP (pure), b) [(1-methyl-1H imidazol-2-yl)sulfanyl] acetic acid (ionic liquid, IL) (modifying agent), c) PVP (pure), d) IL@AuNPs (in the presence of PVP), e) IL@AuNPs + AP (in the presence of PVP).

Supplementary Tables

Table S1. Comparison of sensing performance of AuNPs-based methods for perchlorate detection.

Analytical Method	Sensing material	Linear Range	Detection Limit	References
SERS	AuNPs-Cystaminedihydrochloride	5×10^{-6}-1×10^{-4}mol L^{-1}	5×10^{-6} μmol L^{-1}	(1)
SERS	AuNPs/SiO_2-Various silane reagents	10^{-4} -10^{-5} mol L^{-1}	10^{-6} mol L^{-1}	(2)
SERS	AuNPs-Poly(diallyldimethylammonium chloride)	25 μg/L-50 mg/L	25 μg/kg	(3)
SERS	gold ellipse dimer nanoantenna	0.4-80 μmol L^{-1} (in the absence of interference)	<1×10^{-6} mol L^{-1}	(4)
Spectrophotometry	AuNPs-methylene blue	7.5×10^{-5}-1.0×10^{-3} mol L^{-1}	2.4×10^{-5}mol L^{-1}	(5)
Spectrophotometry	AuNPs- [(1-methyl-1H imidazol-2-yl)sulfanyl] acetic acid- polyvinylpyrrolidone	5×10^{-6}-2.25×10^{-5} mol L^{-1}	1.5 μmol L^{-1}	**This work**

As stated in the literature [6], although the SERS method can detect perchlorate in water in situ and with high sensitivity, the method is not interference-free such that some common anions (e.g. SO_4^{2-}) have adverse effects [4] and equipment costs are high.

References

(1) Ruan, C.; Wang, W.; Gu, B. Surface-enhanced Raman scattering for perchlorate detection using cysteamine-modified gold nanoparticles. *Anal. Chim. Acta*. **2006**, *567*, 114-120. https://doi.org/10.1016/j.aca.2006.01.097.

(2) Wang, W.; Ruan, C.; Gu, B. Development of gold–silica composite nanoparticle substrates for perchlorate detection by surface-enhanced Raman spectroscopy. *Anal. Chim. Acta.* **2006**, *567*, 121-126. https://doi.org/10.1016/j.aca.2006.01.083.

(3) Xiao, J.; Zhang, T.; Li, R.; Meng, Y.; Wen, W. Surface-enhanced Raman scattering for quantitative analysis of perchlorate using poly(diallyldimethylammonium chloride) capped gold nanoparticles. *Applied Spectroscopy.* **2012**, *66*, 1027–1033. https://doi.org/10.1016/j.aca.2006.01.083.

(4) Jubb, A. M.; Hatzinger, P. B.; Gu, B. Trace-level perchlorate analysis of impacted groundwater by elevated gold ellipse dimer nanoantenna surfaceenhanced Raman scattering. *Journal of Raman Spectroscopy.* **2017**, **48**, 518–524. https://doi.org/10.1002/jrs.5070.

(5) Keskin, B.; Üzer, A.; Apak, R. Colorimetric Sensing of Ammonium Perchlorate Using Methylene Blue−Modified Gold Nanoparticles. *Talanta.* **2020**, *206*, 120240. https://doi.org/10.1016/j.talanta.2019.120240.

(6) Hu, J.; Xian, Y.; Wu, Y.; Chen, R.; Dong, H.; Hou, X.; Liang, M.; Wang, B.; Wang, L. Perchlorate occurrence in foodstuffs and water: Analytical methods and techniques for removal from water – A review. *Food Chemistry*. **2021**, *360*, 130146. https://doi.org/10.1016/j.foodchem.2021.130146.
